# Lived experiences of patients with epidermolysis bullosa: A rare genetic skin disease

**DOI:** 10.4102/hsag.v29i0.2824

**Published:** 2024-12-16

**Authors:** Antoinette V. Chateau, David Blackbeard, Colleen Aldous, Ncoza Dlova, Cassidy-Mae Shaw

**Affiliations:** 1 Department of Dermatology, Faculty of Health Sciences, Greys Hospital, Pietermaritzburg, South Africa; 2 Department of Dermatology, Faculty of Health Sciences, University of KwaZulu-Natal, Durban, South Africa; 3 Department of Clinical Psychology, Faculty of Health Sciences, Greys Hospital, Pietermaritzburg, South Africa; 4 Department of Psychiatry, Faculty of Health Sciences, University of KwaZulu-Natal, Durban, South Africa; 5 School of Clinical Medicine, Faculty of Health Sciences, University of KwaZulu-Natal, Durban, South Africa

**Keywords:** epidermolysis bullosa, genetic skin disorder, patients, interpretative phenomenological analysis, impact, quality of life, needs, Africa

## Abstract

**Background:**

Epidermolysis bullosa (EB) is a rare genodermatosis that results in extreme skin fragility, for which there is no cure and may be fatal. The quality of life of patients affected may be greatly impacted.

**Aim:**

This study aims to understand the lived experiences of patients with EB.

**Setting:**

Intensive semi-structured interviews were conducted with three participants via Zoom, and a follow-up member checking session was held in person at the RARE-X conference.

**Methods:**

This qualitative research used interpretative phenomenological analysis with the aim of understanding the lived experiences of patients with EB. Semi-structured interviews were conducted with three participants using Lincoln and Guba’s framework of trustworthiness was used to ensure rigour.

**Results:**

Three adult participants shared in-depth experiences of living with EB. Four themes with subthemes were identified: (1) medical damages, (2) development trajectory, (3) subjective well-being and life satisfaction and (4) sources of resilience and support.

**Conclusion:**

Epidermolysis bullosa affected all developmental stages of life, impacting them physically, emotionally, socially and financially. They shared their concerns relating to a lack of knowledge of healthcare practitioners (HCPs) in managing their illness and society for judging their condition. There is a need for comprehensive biopsychosocial care of patients and their families, as well as continued medical education for HCPs and awareness of society regarding this debilitating condition.

**Contribution:**

To our knowledge, this is the first study in Africa focused on the lived experiences of patients with EB. This highlights the physical, psychosocial and financial challenges that patients with rare diseases encounter in our local setting.

## Introduction

Epidermolysis bullosa (EB) is a rare genodermatosis that results in extreme fragility and blister formation of the skin and mucous membranes. There are four main variants, namely, EB simplex, junctional, dystrophic and Kindler, with over 30 subtypes and 21 known genes (Has et al. 2020). The prevalence and incidence vary with country. The incidence in the United States is 19.57, and the prevalence is 11.07 per 1 million live births (Fine 2016). There is no registry in South Africa; hence, the epidemiology is unknown. Owing to the rarity of the disease and the limited research on the experiences of patients and their families (evident in this article having to reference landmark journal articles older than 5 years), this research is an important contribution to the body of knowledge and implications for services.

Complications of EB include skin infections that may result in septicaemia, skin scarring, skin cancers, scarring of other ectodermal structures such as teeth and nails, gastroesophageal strictures and malabsorption, ocular scarring, musculoskeletal defects, cardiac and renal complications (Fine & Mellerio 2009a, 2009b). There is no cure for EB, and therapy is aimed at preventing blister formation, wound care, treating complications and relieving symptoms (Pope et al. 2012). Diagnostics such as immunofluorescent mapping and genetic testing are not readily available in a resource-limited environment such as South Africa and depend mainly on clinical signs to differentiate the subtypes.

Epidermolysis bullosa has a profound effect on the quality of life of patients and their families and places a strain on family and friendship dynamics (Fine et al. 2005; Martin et al. 2019). The main debilitating symptoms are pain and itch, which can affect patients’ psychological well-being (Schräder et al. 2021). Many people in society have misconceived perceptions as to the aetiology of EB, and this has led to undue judgement and exclusion of many patients with EB (Dures et al. 2011). The health system in South Africa is ill-equipped for patients with rare diseases and complex needs, unlike many other communicable and non-communicable diseases requiring care. Patients have had varied experiences with healthcare practitioners (HCPs), and many left feeling that their needs were not met after the medical encounter (Dures et al. 2011). Kearney, Donohoe and McAuliffe (2020) highlighted the complex needs and challenges of patients with EB, which included the need for informational, psychosocial and physical support; the need for advice regarding benefits and entitlements; and effective interaction with HCPs.

There is a need for HCPs to be aware of these unique challenges and complex needs and to adopt a comprehensive, biopsychosocial and culturally sensitive approach to the management of patients with EB.

## Research methods and design

### Study design

Interpretative phenomenological analysis (IPA) (Smith, Flowers & Larkin 2009) was used to describe and interpret the lived experiences of patients with EB. Interpretative phenomenological analysis is concerned with understanding individual subjective experiences by rendering dynamic, fine-grained accounts of personal perspectives.

The principal investigator’s (PI) interpretative framework is influenced by professional relationships with patients with EB developed through the course of their treatment, previous research in EB (Chateau et al. 2023a, 2023b; Chateau, Blackbeard & Aldous 2023) and experience from specialist paediatric dermatological practice in the area of EB. Therefore, significant efforts were made to ensure collaborative reflection with contributing researchers and regular introspection took place.

### Setting

In-depth semi-structured interviews were conducted with participants (*N* = 3) via Zoom, allowing participants to participate from the comfort of their homes at a time that was convenient for them and their families. A follow-up member checking session was held in person at the RARE-X conference in Johannesburg in February 2024. RARE-X is a rare disease conference that provides a forum for collaboration between patients with rare diseases, doctors and various stakeholders for the purposes of improving diagnostics and possible treatment of rare diseases.

### Study population and sampling strategy

Owing to the very rare nature of EB, purposive sampling was used to access participants who would be able to provide rich information on the phenomenon of the study (Alase 2017; Smith et al. 2009). The limitations of sample size were mitigated by the in-depth interviewing and the fine-grained analysis.

The author approached Dystrophic Epidermolysis Bullosa Research Association South Africa (DEBRA SA) to recruit participants. The president of the association circulated the outline and aim of this study to all members of the organisation. Three individuals responded and were willing to participate. The inclusion criterion was adult individuals with a diagnosis of EB who were willing and consented to participate in the study. The limitations of sample size were mitigated by in-depth interviewing and fine-grained analysis.

Owing to the rarity of the disease and the subsequent small patient population, the demographic details of the participants of this study are restricted to their EB subtype so as to ensure they are not identifiable. Participants have been given pseudonyms to ensure anonymity.

### Data collection

Semi-structured in-depth interviews (Appendix 1) were conducted between September and November 2023 by a postgraduate psychology student trained in the interviewing method. Privacy, confidentiality and comfort were considered for all interviews because of the sensitive nature of the topic. All participants spoke English. Interviews were digitally recorded using the Zoom record function. Interviews ranged between 128 min and 140 min. Debriefing took place between the interviewer and the PI after each interview to adapt questioning and facilitate familiarisation with the data. Interviews were transcribed verbatim by the interviewer. An in-person member-checking meeting was held at a RARE-X conference in Johannesburg in February 2024. Themes and subthemes were shared with the patients to confirm if these were representative of their experiences.

### Data analysis

A collaborative systematic inductive analysis was undertaken by the PI and CS (collaborator) utilising Smith and Nizza’s (2022) four-step process culminating in a framework of experiential themes and subthemes (Table 2).

In an effort to ensure the reliability and validity of the data, Lincoln and Guba’s (1986) framework of trustworthiness in qualitative research and the concepts of credibility, transferability, confirmability and dependability were used to guide the research activity (Lincoln & Guba 1986). A CS was included in the analysis process to ensure that bias and preconceptions did not interfere with or influence the research process. The research team used multiple sources of triangulation, including a third-party interviewer, debriefing, reflective activity, consistent researcher correspondence, maintenance of a thorough audit trail and collaboration in the analysis process. Final integration occurred through reflection and review during the write-up to ensure a clear and accurate presentation of the study and findings.

### Ethical considerations

The study was approved by the Biomedical Research Ethics Committee (BREC/00003768/2022). Informed consent for participation and recording was obtained from participants prior to each interview. All participant data were de-identified and coded for participant confidentiality and anonymity. Stored information was password-protected, with codes and data stored separately. Commitment to sharing study findings with participants was honoured.

## Results

### Demographic characteristics

Three female participants of similar age and who defined themselves as being within the middle-income financial bracket took part in this study (Table 1).

**TABLE 1:** Participants’ characteristics.

Pseudonym	EB subtype
Lee	Junctional EB
Mia	Junctional EB
Niri	Recessive dystrophic EB

EB, epidermolysis bullosa.

Lee was diagnosed with junctional dystrophic EB (intermediate severity) at the age of 2 years. She was employed at the time of the study and lived with friends. Lee felt strongly about patient advocacy. Mia was also diagnosed with junctional dystrophic EB (intermediate severity), was single, lived with family and was employed at the time of the study. Mia was determined to educate peers about EB. Niri was diagnosed with recessive dystrophic EB (severe subtype). She was married and lived with her family. She had been boarded by her previous employer because of poor health. Niri boldly defied boundaries and lived life to the fullest in spite of the severity of her illness.

### Experiential themes

The four major themes and their corresponding subthemes are presented in Table 2.

**TABLE 2:** Experiential themes and subthemes.

Experiential themes	Subthemes
Medical damages	Physical challenges and medical complications
	Difficulties with healthcare services and healthcare systems
	Obligation to be the expert in their own care
Developmental trajectory	An isolated childhood
	Difficulties during the adolescent years
	Experiences during adulthood
Subjective well-being and life satisfaction	Community misunderstanding and the need to educate
	Relationships
	Impact on finances
Sources of resilience and support	Systems of support
	Choice, meaning and purpose

#### Medical damages

Participants reported many physical and emotional challenges navigating life with EB. Their experiences of how the healthcare system and interactions with healthcare professionals created further complications are described next.

**Physical challenges and medical complications:** The core defining features of the participants’ daily experience were the physical challenges and medical complications inherent to having EB, particularly the characteristic blister formation caused by the slightest pressure on the skin, nails and mucosa. Niri spent a considerable amount of time each day attending to her wounds, sharing that EB wounds were unique in that they were constantly in the process of forming and healing and should not be considered analogous to other wounds, such as burns:

‘You really can’t treat it like any other conventional wound healing you have to let it get creative.’ (Niri)

The significant toll of managing physical wounds was demonstrated by Lee, who reported specifically choosing to spend less time on extensive wound care for the sake of her mental health:

‘I would rather spend a little bit less time doing all the bits and bobs and just be like a little mentally happier that I’m not spending four to six hours a day dressing wounds.’ (Lee)

Pain and pruritus are the main debilitating symptoms that result in downtime and absenteeism from daily activities such as school and employment. Niri shared that the itch was so intense that she would tear her skin in an attempt to relieve the itch, only to cause more blisters, setting up an itch-pain-blister cycle:

‘The itch in EB it’s like an itch that’s completely like you are scared like you sometimes think it’s better to be dead … it’s just such deep deep itch it was mentally draining.’ (Niri)

Lee, Niri and Mia shared their daily experience of pain and suffering and were well aware of the systemic nature of EB from their experience of the gastrointestinal tract, gynaecological, ocular, cardiac and musculoskeletal system complications, further exacerbating their pain:

‘Pain is something that is really serious because it’s affecting my heart which I also have chronic pericarditis of the heart so pain is a bit now front and centre.’ (Lee)

Niri shared the daunting realisation that EB was progressive with age and that she was facing more serious health challenges at present:

‘With age it actually you know medically it gets worse it’s not getting better the older I get the worse it’s going to get it’s just taking it day by day.’ (Niri)

**Difficult ies with healthcare services and healthcare systems:** Niri, Mia and Lee were despondent and distressed about health services and the larger health system. The lack of knowledge of HCPs was particularly concerning for participants. They felt that this lack of practitioner knowledge delayed diagnosis (with all three of them having experienced an HCP, assume they were burnt at some stage) and treatment. The participants expressed frustration about specialists who knew little about EB and could not offer differentials and aetiology even after several consults. Lee’s parents’ desperation to find a diagnosis for her at the age of 2 years resulted in her father transporting a skin sample overseas for analysis. Niri only received her exact diagnosis at 26 years of age.

Along with their primary care, other medical care was compromised. Niri was severely injured when the skin was removed during a procedure because of a lack of knowledge of appropriate care:

‘You know, they were pulling the sheet off and they realised that as they pulled all of the skin was coming off with the sheet [.] The anaesthetist fainted, he actually fainted.’ (Niri)

Not only did HCPs lack the required knowledge, but they were also experienced as judgemental and condescending:

‘I’ve had one or two experiences of really just having ignorant arseholes for doctors. I think when you do go in with higher needs and more specific needs you maybe are but more prone to shitty experiences because there are a lot of doctors that feel they’re educated enough and you know kind of categorise you into a box …’ (Lee)

All participants perceived EB as not being prioritised by the healthcare system and negative experiences of the system as a whole. Mia, who accessed public health services, faced the challenges of essential items being out of stock. Niri and Lee noticed the difficulty related to private healthcare services and medical aid schemes, such as not paying for many of the essential medical items for day-to-day care, leaving them to pay out of pocket for these. Medical aids were also perceived as being uninformed and uneducated about appropriate EB care:

‘Medical aid schemes want you to see Joe Bloggs on the network and Joe Bloggs on their network has never seen EB before. EB is not tailored for those medical aids you know.’ (Niri)

In addition to issues with provider options, the participants described how the common 15-min structure of consults was inappropriate, considering the complexity of EB.

The lack of multidisciplinary care for patients with EB across the public and private systems, inclusive of HCPs working in silos, was a key illustrating element of the problematic health system for participants. Although the participants had different circumstances, they shared the difficulty with the transitions between HCPs, which affected the continuity of care and resulted in inadequate follow-up:

‘I don’t complain because first of all I feel like I pay like fifty rand at the government hospital which I’m so thankful for but going to follow ups and knowing you’re not gonna see the doctor that you saw that was so invested. You stress you literally stress out because the doctors are like what did you say you have, I can’t even say the name what is that is that some type of thing with the bones.’ (Mia)

Interestingly, despite the aforementioned, the participants felt more cared for and heard by their GPs than by specialists. Niri shared that her dermatologist had refused to become involved when there had been complications after a procedure.

Lee found that current treatment guidelines were aimed at the affluent and were not adapted to South Africa’s resource-limited health services nor the cultural landscape of South Africa. Lee emphasised the need to be sensitive and aware of varying cultural beliefs when HCPs offer advice regarding the management of patients:

‘It’s difficult to push Western medicine on a family that want to take baby out of hospital to go see a sangoma and wanna go more the traditional route … we have to figure out a way to reach families that are in different cultures and believe different things.’ (Lee)

The participants were very aware of the challenges as well as how improvements could be implemented but were limited in effecting any such change:

‘The difficulty I suppose then of it being rare is there isn’t a lot of us and so it’s very hard to lobby for changes in medical aids and policies and stuff … and it’s even harder for us because we don’t have a database in South Africa … it’s hard to even have an estimation of numbers also because of lack of diagnosis in areas where EB goes completely unrecognised.’ (Lee)

**Obligation to be the expert in their own care:** As a result of systematic inadequacies, participants felt responsible for their own care:

[*T*]he part I struggle with seeing doctors is managing my own care because we don’t have a centre for EB and we don’t have a multidisciplinary team.’ (Lee)

The participants felt that HCPs did not make an effort to research their condition, complications, precautions and appropriate therapy. This resulted in the participants becoming the custodians of their own care, which placed a strain on them in terms of responsibility. Lee recounted that it felt burdensome having to educate a specialist about her condition when attending consultations. She shared wanting to be a part of the decision-making of her care but did not want the pressure and burden of full responsibility for her care:

‘It feels very daunting to be the decision maker … Because EB’s rare and not a lot of people know extensive amounts about it, I wouldn’t necessarily trust somebody else to be calling the shots without my input but at the same time I don’t want to weight of making medical decisions around surgeries and the heart which I have done in recent years.’ (Lee)

#### Developmental trajectory

An inverse trajectory was found in which medical challenges and complications deteriorated with age, although adaptations of development continued with progressive changes.

**An isolated childhood:** The participants reported challenging childhoods and not being able to participate in typical childhood activities.

Participants had not taken part in school sports and extracurricular activities because of the fragility of their skin and instead focused on academic learning. Mia shared that extracurricular activities, such as attending a school camp, had been daunting regarding dressing changes. Sometimes, she had been excluded from fun activities and physical games. Participants identified a constant fear of being injured by others. Mia recalled one such incident when her skin was sloughed off when she had descended a slide while playing with her friends. A unifying concern among all the patients was not wearing a school uniform, which set them apart from their peers:

‘I felt so uncomfortable with myself that I had to wear tracksuits to school I couldn’t even wear a girl’s school uh dress.’ (Mia)

They had felt isolated and spent many lunch breaks alone, watching the other children on the playground:

‘EB being extremely visual, you get one or two compassionate little kiddies that are happy to see a bandage and a plaster … when they’re very young more on their inquisitive side but when they get a little bit older can be on the rude side so yeah many, many break times sitting on a bench by myself.’ (Lee)

**Difficulties during the adolescent years:** Mia, Lee and Niri described their adolescent years as marked by introversion, an awareness of difference, a lack of confidence and self-esteem, bullying and inadequate spaces of support. They felt uncomfortable being wrapped in bandages and were acutely aware of the stark visibility of their disability and inability to live a normal life because of their skin fragility that set them apart:

‘In high school and stuff I was very introverted uhm didn’t just didn’t feel like I could be myself at all … I just couldn’t be the person that I knew I was so boxed in and I just never had the confidence. My self-esteem was very affected in high school because I looked so different I had to wear different uniform people knew there were so many things wrong and different about me and like I felt the weight of having those eyes you know people talking about it like right in front of you all the time all around I had a very small group of friends.’ (Niri)

All participants described having been bullied for being different. Mia shared that she was inaccurately labelled for having other illnesses and that peers had refused to share a bathroom with her:

‘It was bad, I had children throwing rocks at me I had children say you know you’ve got AIDS mixed with cancer … They told me I don’t wanna go in the same bathroom as you do because you’re gonna like affect me.’ (Mia)

Lee recalled that the school day had been met with additional challenges to the usual stressors of the teen years, such as excruciating pain and new blister formation, resulting in the inability to focus and concentrate on the lesson at hand:

‘Going to school … just meant pain, it meant I’m not in my comfortable home where if something hurts me I can just put my feet up or I can stop or I can ask for help or whatever uhm so I think it was physically quite painful which then translated to emotional stress uhm and anxiety.’ (Lee)

The participants agreed that mainstream schools were not equipped to meet the needs of students with physical challenges:

‘Just to be in mainstream schooling Monday to Friday was a fat task because you’re trying to put a kid with high needs into mainstream and it’s not adaptable enough so we learned coping mechanisms. I don’t think it was an enjoyable time.’ (Lee)

Owing to inadequate inclusive education support, Mia was placed in a special needs school because of her school performance challenges having been inappropriately attributed to low intellectual functioning when they actually resulted from interruptions in her learning caused by her physical condition. The inappropriateness of this assessment was only established later on after having lost many years in mainstream schooling, which impacted her subject choice and, ultimately, career choice.

**Experiences during adulthood:** A key experience in adulthood was coming into one’s own despite challenges. Lee and Niri described having gained confidence and developing their ability to form meaningful friendships and relationships:

‘I only really grew the confidence after I left high school … I had so many friends that were older that didn’t worry about things and it really gave me a little boost of confidence … I only really started dating when I got to university it was when I had my first boyfriend, and then my husband. I was lucky that he was a good guy.’ (Niri)

Although their self-confidence had improved in adulthood, they were all very conscious and concerned about their physical deterioration and the challenges regarding work and employment.

Epidermolysis bullosa was perceived to have far-reaching effects on career outcomes, primarily because of frequent absenteeism caused by disease flares or complications. Mia, Lee, and Niri shared that the work environment, like school, was not adapted to meet the needs of people with the physical challenges that they faced nor the associated mental challenges. They shared that EB-friendly jobs were low-paying and inconsistent with the patient’s passion or career goals:

‘There are not enough availability for jobs for people that need adjusted working conditions so you either then end up doing something that is not your passion but is EB friendly and pays well or you end up doing adjusted position but earning a quarter of the salary of somebody else that isn’t in an adjusted position. So it’s trying to juggle what you would like to do with your life like anyone else wants … and you also need to be able to pay medical bills and put food on the table and start a life and you know so uhm that has been a bit of a struggle.’ (Lee)

Mia had experienced strain because the job market was not empathetic to health challenges. The difficulties she faced with attending hospital appointments because of a no-work-no-pay policy provoked such anxiety that resulted in an admission to a mental health facility:

‘I literally had applied for eighty-three jobs last year only got one interview and that was with [*company*] I’m not happy I am honest, I hate my job. I hate it to the point where I’m like getting anxiety attacks … Did I ask to become sick? It happens because my mental every everything’s taking its toll it’s too much [.] and people just don’t understand that like you’re a liability for the company so bye.’ (Mia)

Lee similarly felt unsupported in her workspace to the extent of feeling constant pressure to ‘keep up with the pace’ despite her significant physical challenges.

#### Subjective well-being and life satisfaction

Epidermolysis bullosa had far-reaching effects on the participants, impacting their quality of life.

**Community misunderstanding and the need to educate:** All participants had experienced people around them misunderstanding their condition, experiencing other people staring at them and making inappropriate comments and offering unfounded advice that was unhelpful and, in some instances, harmful. There was a lack of community support felt by the participants, with those around them even assuming that they had mental challenges because of their physical disability:

‘The first big misconception was and I remember people saying this from the time I was small was oh she’ll grow out of it. I got told I should blend frozen cucumbers, it’s going to make the blisters go away. There’s no truth in it and it adds no value to the situation because the parents … know it’s not going to get any better in fact actually it’s going to get worse.’ (Niri)

Lee shared an encounter at an airport where a staff member was directing all questions to the person accompanying her because ‘she saw the wheelchair and I think she also thought my brain was a potato’.

Lee, Niri and Mia all felt obligated to educate their respective communities about their illnesses from a young age and took it upon themselves to do displays for their classmates and talk to their peers at school. The participants detailed how having to engage in such activities and conversations, while useful because it allowed others to ask questions and dispel myths, took a toll on their experience of daily life, having not received support from those around them and having to educate those around them continuously.

**Relationships:** Having EB presented significant challenges in terms of relationships and had a profound influence on the participants’ perceptions of themselves and their ability to play various relational roles. Niri aptly described feeling ‘on the sidelines’ and ‘less than’ in relation to those around her. A shared difficulty amongst the participants was the reliance inherent to relationships because of the difficulties related to living independently. They all shared that they received immense support from their parents but had varied experiences regarding sibling support. Lee shared that her sister was ‘not the most empathetic person’, while Niri found her sister to be highly supportive to the extent of denying herself opportunities in activities that Niri could not participate in. Despite this, Niri acknowledged that the responsibility placed on the family to care for a relative with EB ‘really holds the other sibling back’.

Niri was happily married to a supportive husband, having gained confidence and openness to relationships at a young age, which allowed her to be confident and direct with her partners about her condition. Lee, on the other hand, held back in relationships and felt like a burden, holding the assumption that the condition would be too difficult for a partner to manage. She found it challenging to comprehend how a partner would be attracted to her wounds, even expressing the following:

‘I just generally often think that I am a lot of baggage and you know, I need a lot of care a lot of help and I cost a lot so whenever any sort of relationship … I think that they could potentially do a lot better than me because my EB is degenerative and I notice that over the years my capacity is less for many things so I will develop feelings and then think no this person could definitely do better.’ (Lee)

Niri shared that EB was a significant aspect of her relationship with her children, fearing that they would be bullied because of her condition, and found herself focusing a lot of their relationship on sensitising them to EB and empowering them to teach others about the condition.

Mia and Lee felt hindered from taking on the role of mother because of concerns and doubts about the practicality and timing of motherhood and giving birth as well as fears about passing the condition on. The financial and physical implications of raising children were also perceived as limiting factors that held them back from this type of relationship. Mia described the psychological impact of her medical condition precluding her from being a mother as a ‘setback’ and pondered that she ‘will probably be alone till the day I die’. Lee felt there was no time for her to engage in the relational aspects of life:

‘I do think EB’s clock ticks a bit faster … because my EB is degenerating, I think I’m not as young as my actual age.’ (Lee)

**Impact on finances:** All three participants shared that their care placed a huge financial burden on them. Medical care and dressings cost exorbitant amounts, and the participants could not afford EB-appropriate dressings, often having to reuse dressings and needles because of the major disparity between socio-economic groups. Not all medical aids or health insurance covered the necessary items, forcing participants to rely on family for assistance with medical items, and adapting their home and making it more accessible and functional, extending the burden to their family and caregivers. The participants experienced employers as unsupportive of employees with medical needs. They faced the reality of no work no pay, often having to attend work despite being unwell to earn a day’s wage. Niri shared that she had been medically boarded after numerous health challenges and remained unemployed, which had dramatically impacted her financial security.

#### Sources of resilience and support

Despite the various challenges experienced by the participants, they were able to tap into their inner strength and resilience.

**Systems of support:** Lee, Niri and Mia shared the importance of their faith in God and the support of family and friends. Support structures such as DEBRA SA and Rare Diseases South Africa empowered participants with the knowledge and provided support and assistance to families and persons living with EB in need. Epidermolysis bullosa support groups and educational material available helped the participants to dispel myths about the condition, change how society perceives people with EB and empower families to deal with the condition.

**Choice, meaning and purpose:** Niri, who suffered from the most severe subtype of EB, was full of confidence and did not let her condition deter her from living life to the fullest. She made a concerted effort to enjoy her life and push the boundaries. She adapted to her condition and did not let EB to stand in her way, noting:

‘Actually, people don’t really worry about the skin once you are somebody that’s confident.’ (Niri)

Lee found purpose in reaching out to other families who were affected by EB. She felt able to understand the journey others were going through based on her tough experience of getting a diagnosis and treatment. She felt driven to make other families’ lives easier where she could.

Mia had faced prejudice, which had far-reaching effects on her academics, work opportunities and income generation. She soldiered on, finding passion in educating others about EB, and was able to obtain a stable, permanent, yet challenging job.

## Discussion

Epidermolysis bullosa has profoundly affected the lives of the participants, and this study highlighted similar findings in previous studies related to persons with rare diseases (Sangha, MacLellan & Pope 2021) that they face uncertainty about the future; the physical and emotional challenges associated with the progression of the disease; a lack of therapy; concern around the lack of knowledge of HCPs; a lack of continuity of care; and the enormous responsibility placed on patients to take on the role of expert that leads to potential patient burnout. Despite the challenges, the participants in this study were able to find the courage to adapt their lives as a result of the support structures around them.

The physical symptoms of pain and pruritus consumed all facets of our participants’ lives. Pruritus can be debilitating, setting up an itch-scratch cycle that results in new blister formation, disrupted sleep, and decreased concentration and productivity (Mauritz et al. 2019; Van Scheppingen et al. 2008). Pain may be related to bathing, dressing changes, injury, surgical procedures or complications. The severity of the pain is usually congruent to the severity of the subtype of EB (Schräder, Yuen & Jonkman 2018). In addition to the primary symptoms, complications experienced by participants were infections, oesophageal stenosis, ocular scarring, cardiac, orthopaedic and gynaecological concerns, similar to findings in the literature (Fine & Mellerio 2009a). Participants expressed concern about increased complications and their deteriorating health with age. These debilitating symptoms and complications had far-reaching effects on school performance, the opportunity for tertiary studies, career choices and, ultimately, finances.

Martin et al. (2019) reported that dressing changes are lengthy and painful, placing great strain on patients and families assisting with care. Some participants in our study spent a considerable length of time performing dressing changes, whereas others chose to manage their wounds sub-optimally in an effort to gain control (Chernyshov et al. 2024) and for the sake of their mental health. Previous studies have highlighted the layered struggles patients endure during dressing changes, including loss of spontaneity and independence, decreased self-esteem and the negative impact on emotional state (Grocott et al. 2013; Sangha et al. 2021).

Little is known about EB, which delays diagnosis and management (David et al. 2023). All participants struggled with timely diagnosis, with one family even travelling abroad with her skin sample for an expert opinion. All the participants had negative experiences with HCPs, reporting that they felt that HCPs were not proficient with their condition and did not have knowledge of the long-term effects of their condition. Some HCPs disclosed to the participants that they had never heard of EB before and that no effort had been made to learn more about the disease for follow-up consultations. Participants shared that HCPs were judgemental and assumed that they had been burnt. Dures et al. (2011) found that patients suffered physically and psychologically as a result of ignorance of HCPs. Interestingly, all participants felt more supported by their general practitioners than their specialists, noting more assistance, continuity and invested care, similar to the findings by Lewis et al. (2000). They shared their concern that HCPs work in silos with a lack of continuity of care and suggested that multidisciplinary teamwork is essential for comprehensive care. This was similar to suggestions by Martin et al. (2019) who stated that HCPs should work in a multidisciplinary team for the optimal outcome of patients. The participants experienced a significant burden and responsibility in assuming the role of the expert, informing HCPs about their condition and monitoring their care. Other studies have shown similar findings where HCPs lacked knowledge of the condition, families felt ignored by the HCP team and HCPs were paternalistic and not open to dialogue (Budych, Helms & Schultz 2012; Kearney et al. 2020; Van Scheppingen et al. 2008). Budych et al. (2012) found that collaboration between patient and HCP and good physician–patient interaction were essential for patient care. In contrast, confrontational interactions, wherein physicians exert control and ignore patients’ suggestions or input, lead to poor rapport.

An inverse trajectory was observed among our participants, with an increase in confidence and the formation of meaningful relationships despite declining health with age. All participants shared the difficulty adapting in their school-going years. They felt alone and isolated in primary school, sat out of sports and recreational activities, and had to navigate mainstream schooling that was not adapted to their needs and health challenges (Sangha et al. 2021; Van Scheppingen et al. 2008). Studies have found that patients with EB have difficulty joining peers and have to continuously weigh up the risk-benefit ratio when doing activities, often driven by not wanting to be left out (Dures et al. 2011; Van Scheppingen et al. 2008). High school was characterised by bullying, self-consciousness and a lack of confidence. Van Scheppingen et al. (2008) reported that the visibility of EB resulted in patients being bullied and accused of being contagious, teased and stared at, similarly experienced by our participants. Participants grappled with being visibly different from their peers, as found by Williams, Gannon and Soon (2011) who highlighted their study participants seeing themselves as ‘wrong’ and experiencing negative experiences with peers. Chernyshov et al. (2024) reported that patients with skin conditions who were victims of bullying might have long-term social and psychological consequences and a higher rate of social issues, psychosomatic problems, depression, anxiety disorders and suicidal ideations. Our participants experienced some of these consequences at various stages of their lives.

Participants observed that society made assumptions that their physical challenges have associated mental challenges, which resulted in one of the participants being incorrectly placed in a special needs educational setting. This had far-reaching effects on her schooling and, consequently, career choices and financial well-being. Sangha et al. found similar findings in which children with EB were treated differently, and assumptions were made that they had learning disabilities (Sangha et al. 2021). Participants seemed to ‘blossom’ after leaving high school, adapting to their disease, gaining confidence and forming meaningful romantic relationships. However, increased physical challenges and complications requiring increased specialist consultation with age marred this upward personal growth trajectory.

A significant burden was placed on the participants to educate society about EB because of the stigma of the condition. Society was judgemental and made assumptions about participants and their condition, assuming that they had been burnt (similar to how HCPs did) and that the condition was contagious. Similar to the findings of Sangha et al. (2021), many in the community would make inappropriate suggestions that they would outgrow their genetic conditions and offer advice about cures.

Dures et al. (2011) noticed that the stigma associated with the visibility of EB and unpleasant reactions from society resulted in patients developing coping strategies, including asking people who stared at them if they had questions about their condition in an effort to decrease stigma. Participants in our study developed similar coping strategies and made plans to address their classmates at school; they advocated for their care, liaising with medical aids to campaign for expense coverage.

Epidermolysis bullosa places an enormous financial burden on patients and their families. The job market is not geared towards patients with high medical needs and challenges. Thus, participants indicated choosing EB-friendly jobs that they have no passion for and do not pay well but are empathetic to their health challenges. Work proved highly challenging for all participants, with workplaces lacking empathy, resulting in pay cuts and loss of employment, and EB-friendly jobs were infrequent.

Expensive dressings and medical expenses are unaffordable to many EB patients. Angelis et al. (2016) found that patients with EB had a lower health-related quality of life, with substantive direct and indirect costs with socio-economic implications. Some participants depended on public healthcare and did not have the necessary dressings. Jacobson (2022) found that rare diseases are not a priority in the public healthcare sector, medications are unavailable and resources are limited. Jacobson (2022) further noted that rare diseases are chronic and should be covered in full under Prescribed Minimum Benefits (PMB) in the private healthcare sector, which it is not. This was the experience of the participants in the study who had private medical insurance yet had to pay out of pocket for many essentials not covered by their scheme. Participants found that medical aid schemes suggest network providers that may not have experience in rare diseases such as EB and that they lacked a choice in the HCP that they could consult.

All participants needed assistance with dressings, making them reliant upon assistance from others. A study by Fine et al. (2004) reported that patients with different variants of EB had various extents of dependence on others, all requiring assistance at some point. Relationships presented significant challenges. Studies have shown that parents spend less time with their other children while focusing on the child with a rare disease (Plumridge et al. 2011; Van Scheppingen et al. 2008), which may strain sibling relationships. Plumridge et al. (2011) found that some siblings of patients with genetic conditions were resentful or withdrew from the affected sibling, being uncertain how the condition could affect them.

A contrary association between subtype severity and confidence level among the participants was observed. The participant with the most severe subtype of EB expressed feeling confident in her skin as an adult, made friends easily, had meaningful relationships and now has a family of her own. The other participants made friends easily but lacked confidence and were hesitant about meaningful romantic relationships and motherhood.

The participants reported that despite their complex health challenges, they had adapted to their condition and were trying to make the most of life.

Patients shared the benefit of faith/spirituality and support from family, friends and organisations such as DEBRA and Rare Diseases South Africa. Being part of an organisation empowered them with knowledge of the disease, a sense of acceptance, belonging and support to cope. It is evident that there is a need for better medical and social/financial support. Butterworth et al. (2019) noted that improved quality of life for patients with EB requires social, physical, medical, psychological, financial and educational support.

### Limitations

The small sample was limited by availability and convenience because of the absence of an epidermolysis registry in South Africa. However, an integrated account of the patient’s experience was achieved within the limitations of the sample.

### Recommendations

Centres of excellence should be established for rapid referral, diagnostics, prevention and management of complications and multidisciplinary care of patients. There should be continuous education provided to HCPs to ensure rapid diagnosis and referral to specialist centres. This education should be available to societies, communities and schools to prevent stigma and bullying and foster acceptance of patients with EB.

Schools should cater and be adapted for children with high needs and disabilities. Children with EB should be integrated into mainstream schools that are cognisant of and accommodate their limitations.

Continuity of care in the healthcare sector is vital to ensure effective and comprehensive care of patients, which will decrease anxiety among patients. An effective transition of care is essential when moving between paediatric and adult healthcare services.

Home-based care should be available to assist patients with severe disease to limit numerous trips to healthcare services that potentially put them at further risk of injury, infection and progression of their disease.

Rare diseases should be prioritised within the healthcare sector and social agencies in South Africa. There should be social or financial support via the South African Social Security Agency for a care dependency grant if the patient is a child or a disability grant if the patient is an adult, as well as medical support for families with rare diseases who may be in need.

Medical aids should be held accountable, and EB should be covered under PMB to provide essential dressings and patient care.

Pharmaceutical companies should be held accountable to make treatment affordable and accessible to all. The job market should cater to and be adapted for patients with special needs.

There should be ongoing biopsychosocial support for patients and families to ensure comprehensive care of patients.

An EB registry should be developed to establish the incidence and prevalence of EB in South Africa. The authors are in the process of developing guidelines for the comprehensive care of patients.

## Conclusion

Epidermolysis bullosa is a rare genetic skin condition that profoundly affects all the participants across all developmental stages of life, impacting them physically, emotionally, socially and financially. They shared their concerns relating to a lack of knowledge of HCPs in managing their illness and society judging their condition. There is a need for comprehensive biopsychosocial care of patients and their families, as well as continued medical education for HCPs and awareness of society regarding this debilitating condition.

## Appendix 1: Semi-structures in-depth interviews with people living with epidermolysis bullosa

1. Effect of living with epidermolysis bullosa (EB) on impact and quality of life:
  - How has EB influenced your education and schooling?
  - How has EB influenced your finances?
  - How has EB influenced your family relationships?
  - How has EB influenced your social relationships (friends, neighbours, colleagues)?
  - How has EB influenced your relationship with a partner?
  - How has EB influenced your occupational activities (work, employment, income generation)?
  - How has EB influenced your relationship with society?
  - How has EB impacted your quality of life? (your general experience in all areas of life)
2. What are the main symptoms that trouble you and how do you cope?
3. What was more or less effective?
  - What dressings were the most effective?
  - What helped to prevent blister formation?
4. Healthcare practitioners and hospitals:
  - Please tell me about your experience of doctors/nurses in relation to EB?
  - What general challenges have you experienced with healthcare for EB?
  - What specific challenges or difficulties have you experienced in accessing or receiving care?
  - What challenges have you experienced at healthcare facilities?
  - How would you like to be involved in the decision-making processes regarding EB care?
5. What are the perceptions in the community about EB?
6. What are the support structures for living with EB?
7. How has living with EB influenced any decisions about having children?
8. What physical challenges did you experience in relation to EB?
9. We are formulating EB guidelines of care, what would you like to see be covered in this document?
10. What healthcare services would be ideal?
